# Secukinumab May Be an Effective Treatment Option for Axial Spondyloarthritis and Psoriatic Arthritis Patients with a History of Malignancy: Multicenter Real-Life Experience from Turkey

**DOI:** 10.3390/jcm13206216

**Published:** 2024-10-18

**Authors:** Tuğba Ocak, Burcu Yağız, Birol Ocak, Özge Yoğurtçu, Fatma Başıbüyük, Dilek Tezcan, Selime Ermurat, Elif İnanç, Gülşah Yamancan, Fatih Albayrak, Rabia Pişkin Sağır, Ayşe Nur Bayındır Akbaş, Osman Cüre, Belkıs Nihan Coşkun, Servet Yolbaş, Uğur Karasu, Bünyamin Kısacık, Süleyman Serdar Koca, İsmail Sarı, Servet Akar, Ediz Dalkılıç, Yavuz Pehlivan

**Affiliations:** 1 Division of Rheumatology, Department of Internal Medicine, Faculty of Medicine, Uludağ University, 16285 Bursa, Turkey; burcuyilmaz_84@hotmail.com (B.Y.); belkisnihanseniz@hotmail.com (B.N.C.); edizinci@hotmail.com (E.D.); drypehlivan@gmail.com (Y.P.); 2 Department of Medical Oncology, Bursa Yüksek İhtisas Training and Research Hospital, 16140 Bursa, Turkey; birol08ocak@gmail.com; 3 Division of Rheumatology, Department of Internal Medicine, Faculty of Medicine, İzmir Katip Çelebi University, 35620 İzmir, Turkey; hatipogluozge91@gmail.com (Ö.Y.); servet.akar@gmail.com (S.A.); 4 Division of Rheumatology, Department of Internal Medicine, Faculty of Medicine, Dokuz Eylül University, 35340 İzmir, Turkey; mdfatmabasibuyuk@gmail.com (F.B.); ismailsari35@gmail.com (İ.S.); 5 Division of Rheumatology, Department of Internal Medicine, Gülhane Faculty of Medicine, University of Health Sciences, 06018 Ankara, Turkey; dr_dilekturan@hotmail.com; 6 Department of Rheumatology, Bursa Yüksek İhtisas Training and Research Hospital, 16140 Bursa, Turkey; selimeermurat@hotmail.com; 7 Division of Rheumatology, Department of Internal Medicine, Faculty of Medicine, İnönü University, 44000 Malatya, Turkey; elif.temelli@hotmail.com (E.İ.); servetyolbas@yahoo.com.tr (S.Y.); 8 Division of Rheumatology, Department of Internal Medicine, Faculty of Medicine, Fırat University, 23119 Elazığ, Turkey; gulsahaydn@windowslive.com (G.Y.); kocassk@yahoo.com (S.S.K.); 9 Department of Rheumatology, Gaziantep City Hospital, 27470 Gaziantep, Turkey; drfalbayrak@yahoo.com; 10 Department of Rheumatology, Bitlis Tatvan State Hospital, 13200 Bitlis, Turkey; piskinrabia@hotmail.com; 11 Division of Rheumatology, Department of Internal Medicine, Faculty of Medicine, Pamukkale University, 20070 Denizli, Turkey; draysenurbayindir@gmail.com (A.N.B.A.); u_karasu@yahoo.com (U.K.); 12 Division of Rheumatology, Department of Internal Medicine, Faculty of Medicine, Recep Tayyip Erdoğan University, 53100 Rize, Turkey; creosman61@gmail.com; 13 Department of Rheumatology, Sanko University Medical Faculty Hospital, 16049 Gaziantep, Turkey; bunyamin.kisacik@yahoo.com

**Keywords:** axial spondyloarthritis, psoriatic arthritis, malignancy, secukinumab

## Abstract

**Background**: Secukinumab is a monoclonal antibody against interleukin 17 approved for patients with axial spondyloarthritis (axSpA), psoriatic arthritis (PsA), and psoriasis. Treating axSpA and PsA patients with a history of malignancy is a challenge. While initial results on the applicability of secukinumab in this patient group are positive, the number of studies on this topic remains limited. This study aimed to investigate the drug’s survival time and the efficacy and safety of secukinumab treatment in this specific patient group. **Methods**: This retrospective study included 30 patients with a history of malignancy who were followed up in rheumatology outpatient clinics in 12 centers throughout Turkey and treated with secukinumab between May 2018 and March 2024 with a diagnosis of axSpA and PsA. **Results:** The mean follow-up time was 29.8 ± 19.3 months. The drug retention rate was 89.7% after 12 months and 80.6% after 24 months. The most common tumor in our study was papillary thyroid carcinoma (*n* = 5, 16.7%). During follow-up, local tumor recurrence was observed in a patient with urothelial carcinoma of the bladder. **Conclusions**: In the largest cohort reported to date, treatment with secukinumab in axSpA and PsA patients with a history of malignancy was not shown to cause oncologic recurrence except for one local tumor recurrence. Drug retention rates were also high, and disease activation and function improved compared to baseline. Therefore, secukinumab could be a safe and effective option for this patient group.

## 1. Introduction

Axial spondyloarthritis (axSpA) and psoriatic arthritis (PsA) are chronic inflammatory diseases within the spondyloarthritis group [1,2]. Nonsteroidal anti-inflammatory drugs and tumor necrosis factor (TNF) inhibitors have been used for many years to treat these diseases in cases of resistance to conventional treatments [3,4]. In recent years, interleukin-17 (IL-17) inhibitors have become an alternative treatment to TNF inhibitors and are also used as first-line biological disease-modifying antirheumatic drugs (bDMARDs). Secukinumab, a fully human immunoglobulin G1 monoclonal antibody targeting IL-17A, has been shown to improve patient symptoms and signs [5]. Our country approved it in May 2018 for treating axSpA and PsA.

When AxSpA and PsA patients have a history of malignancy, treatment management is challenging due to concerns about the impact of biological therapies on cancer recurrence and progression. Although the results are conflicting, TNF and Janus kinase inhibitors should be used with caution in the presence of malignancy as they may affect oncologic prognosis [6,7]. In particular, studies have reported that about half of the patients using biological agents in real life would not meet the inclusion criteria of pivotal randomized controlled trials [8]. A history of malignancy is one of the exclusion criteria in randomized controlled trials. Therefore, the description of the use of biologics such as secukinumab in patients with a history of malignancy is of utmost importance. Although there are few studies on secukinumab, studies have shown that long-term treatment is not associated with an increased risk of cancer development [9,10]. No cancer recurrence was observed in a study conducted with psoriasis patients with malignancy [11]. Similarly, in a study of 22 SpA patients with a history of malignancy, no tumor recurrence was observed [12]. Interestingly, many studies have even found that IL-17A plays an important role in developing various types of cancer [13,14,15]. The inhibition of IL-17 in animal models has shown promising results in preventing tumor progression [16].

AxSpA and PsA patients with a history of malignancy constitute a special population that requires follow-up for both malignant and rheumatologic diseases. Studies on practical experience in this area are limited to a few case series and small patient groups [12,17]. Our multicenter study aimed to investigate the malignancy prognosis, survival, and treatment efficacy of secukinumab in axSpA and PsA patients with a history of cancer.

## 2. Materials and Methods

### 2.1. Data Source and Study Design

Twelve rheumatology centers in Turkey participated in this retrospective study. These centers treated 947 outpatients aged 18 and above, diagnosed with axSpA or PsA according to the Assessment of SpondyloArthritis International Society (ASAS) [18] and the Classification Criteria for Psoriatic Arthritis (CASPAR) [19], with secukinumab between May 2018 and March 2024. The status of the primary tumor (T), regional lymph nodes (N), and distant metastases (M) in solid organ malignancies was determined using the TNM classification of the American Joint Committee on Cancer (AJCC) [20]. Lymphoma staging was performed according to the Lugano classification [21]. Patients with AxSpA received 150 mg secukinumab subcutaneously (once a week for four weeks, then every four weeks), and patients with PsA received 300 mg (once a week for four weeks, then every four weeks). The patients’ data were analyzed retrospectively via the hospitals’ electronic system.

### 2.2. Study Population

Thirty-seven patients were diagnosed with malignancy before secukinumab treatment. Patients were included in this study if they had a history of malignancy, were over 18 years of age, had been treated with secukinumab for axSpA or PsA for at least six months, and had an outpatient follow-up every three months. Seven patients whose duration of treatment with secukinumab had not yet reached six months and who switched to another center for follow-up were excluded from the study. A total of 30 patients were therefore included in the study. Patients who had not received biologic therapy before treatment with secukinumab were defined as bionaive, and patients who had received biologic therapy before treatment with secukinumab were defined as bioexperienced. Both bionaive and bioexperienced patients were included in this study.

### 2.3. Study Outcomes

Gender, age, body mass index, comorbidities, human leukocyte antigen (HLA) B27 positivity status, number of previous biologic therapies for axSpA and PsA, type of biologic therapies, pathologic information, diagnostic stage, and type of cancer treatment were reviewed. The primary endpoint of this study was tumor recurrence and a new cancer diagnosis during treatment with secukinumab. The primary endpoint was assessed at 3-, 6-, and 12-month visits. The secondary endpoint of this study was the duration of treatment with secukinumab and the efficacy of the treatment. The effectiveness of secukinumab was assessed by erythrocyte sedimentation rate (ESR), C-reactive protein (CRP), 10 cm visual analog scale (VAS) [22], Bath Ankylosing Spondylitis Disease Activity Index (BASDAI) [23], Bath Ankylosing Spondylitis Functional Index (BASFI) [24], and Ankylosing Spondylitis Disease Activity Score (ASDAS) CRP [25] in axSpA and PsA patients with axial involvement. Disease Activity Score-28 (DAS 28) CRP [26] markers were used in PsA patients with only peripheral involvement. The secondary endpoint was assessed at 3-, 6-, and 12-month visits. The survival of secukinumab was quantified as the time from the start of secukinumab until the end of treatment (definitive discontinuation) or until the end of data collection in those patients who continued on treatment. Treatment persistence was defined as information in the retrospectively reviewed outpatient clinic records, showing that the patient was treated with secukinumab at the last outpatient visit and that a secukinumab prescription was issued.

### 2.4. Statistical Analysis

Statistical analysis was conducted using SPSS (Statistical Package for Social Sciences) version 26.0. Categorical and continuous variables are reported as absolute numbers, percentages, and mean with standard deviation, respectively. The normality of variables was assessed using the Shapiro–Wilk and Kolmogorov–Smirnov tests. The Wilcoxon test was used to compare the change in BASDAI, BASFI, ASDAS CRP, and VAS between the treatment start and the month 12 visit. A paired-samples test was used to compare the change in ESR between the treatment start and the month 12 visit. Kaplan–Meier analysis was used to study the survival of secukinumab.

## 3. Results

### 3.1. General Characteristics of Patients

Of the 30 patients included in this study, 20 (66.7%) were male. Their demographic and rheumatologic characteristics are shown in Table 1. The number of patients who were currently smoking and drinking alcohol was four. In 16 patients, comorbidities accompanied the malignancy, and hypertension was the most common comorbidity (*n* = 8, 26.7%). The age at diagnosis of AxSpA or PsA was 42.4 ± 11.5 years; 20 (66.7%) patients had AxSpA, 8 (26.7%) had PsA with axial involvement, and 2 (6.6%) had PsA with peripheral involvement. Nineteen of the patients were bioexperienced. Ten used two different biologics, eight used one biologic, and one used four different biologics. The most commonly used biologic among experienced patients was adalimumab (*n* = 12, 63.2%).

### 3.2. Malignancy Characteristics of Patients

The characteristics of the tumors and the treatments applied are listed in Table 2. The most common tumor was papillary thyroid carcinoma (*n* = 5, 16.7%), followed by invasive ductal carcinoma of the breast (*n* = 4, 13.3%), renal cell carcinoma (*n* = 3, 10%), and prostate adenocarcinoma (*n* = 3, 10%). Patient 16 had three primary tumors (adenocarcinoma of the colon, renal cell carcinoma, and papillary thyroid carcinoma), and patient 18 had two primary tumors (adenocarcinoma of the lung and basal cell carcinoma). The most common treatment was surgery (*n* = 28, 93.3%). Twenty-nine patients were in remission when treatment with secukinumab was started. Patient 30, who had differentiated thyroid carcinoma, was being treated with sorafenib for progressive disease at the time of initiation of secukinumab treatment. The median number of months between the diagnosis of malignancy and the start of treatment with secukinumab was 27.5 (2.7–21.8) months. During treatment with secukinumab, no tumor recurrence and no new cancer were observed at 3, 6, and 12 months. During follow-up with secukinumab, a local recurrence was detected in patient 25 after 51.5 months of secukinumab treatment, and the patient underwent reoperation.

### 3.3. Drug Retention Rates and Effectiveness of Secukinumab Treatment

The mean follow-up time of all patients was 29.8 ± 19.3 months, and the median was 25.3 (7.1–71.3). The drug retention rates of the patients amounted to 89.7% after 12 months and 80.6% after 24 months. The median follow-up time was 26.9 months (9.2–58.4) for bionaive patients and 23.7 months (7.1–71.3) for bioexperienced patients. The secukinumab dose administered was 150 mg for patients with axSpA (*n* = 20, 66.7%) and 300 mg for patients with PsA (*n* = 10, 33.3%). Secukinumab treatment was discontinued in five patients due to ineffectiveness; four were bioexperienced. No significant drug-related adverse events were observed in any patients. The duration of secukinumab follow-up for the discontinued patients (patient 6, patient 17, patient 22, patient 23, and patient 28) was 7.1, 11.7, 9.2, 23.7, and 13.2 months, respectively.

When treatment efficacy was assessed after 3 months, ESR was 23.5 (3−70) mm/h, CRP 6.7 (2–30) mg/L, BASDAI 4.2 (1.4–8), BASFI 2.7 (1–7.5), VAS 6 (2–9), and ASDAS CRP 3.3 (1.4–4.17); after 6 months, ESR was 20 (6–56) mm/h, CRP 5.25 (2–23) mg/L, BASDAI 2.7 (1–7.5), BASFI 2.1 (1–24), VAS 4 (1–9), and ASDAS CRP 2.2 ± 0.7; and ESR was 20 ± 9 mm/h, CRP 5 (2–46) mg/L, BASDAI 1.8 (1–6.6), BASFI 1.4 (1–6.9), VAS 2 (0–5), and ASDAS CRP 1.3 (1.1–2.8) at 12 months. The comparison of inflammatory markers and disease activity between the secukinumab baseline and the month 12 visit is shown in Table 3. The ESR, CRP, and VAS values of all patients significantly decreased in the 12th month of secukinumab treatment compared to the baseline visit (*p* < 0.001, *p* = 0.001, and *p* < 0.001, respectively). The ASDAS-CRP scores of patients with axial involvement decreased from 4.3 (range: 3.1–5.2, indicating very high disease activity) to 1.3 (range: 1.1–2.8, indicating remission), and BASDAI scores decreased from 6.2 (range: 5.4–8.2, indicating active disease) to 1.8 (range: 1–6.6, indicating inactive disease). Patients with axial involvement, BASDAI, BASFI, and ASDAS-CRP scores decreased significantly at the month 12 visit following secukinumab treatment compared to the baseline visit (*p* < 0.001, *p* < 0.001 and *p* < 0.001, respectively). There were two PsA patients with peripheral involvement. When evaluating the DAS28-CRP values at baseline and the month 12 visit in these patients, the DAS28-CRP value decreased from 5.2 (indicating very high disease activity) to 2.7 (indicating low disease activity) in one patient and from 5.5 (indicating very high disease activity) to 3.1 (indicating low disease activity) in the other patient.

## 4. Discussion

This study is the largest real-life experience with axSpA and PsA patients with a history of malignancy. No recurrence was observed during the median follow-up time of 29.8 months, except for local tumor recurrence in one patient. Local tumor recurrence has been observed in early-stage urothelial carcinoma of the bladder. The rate of local tumor recurrence is higher in this early-stage cancer than in many other cancers [27]. Significant improvements in disease activity and functional status were observed, as evidenced by decreased disease activity markers and laboratory parameters. These results suggest that treatment with secukinumab may be a good option for this particular patient group with difficult treatment management.

Studies of patients with a history of malignancy treated with secukinumab have generally been conducted with a small number of patients. The study which had the largest number of patients on this subject was conducted with 44 psoriasis patients. In that study, by Pellegrini et al., no tumor recurrence or new tumor development was observed during treatment with secukinumab, although the mean follow-up time was short [12]. In a study by Tekgöz et al. in SpA patients with a history of malignancy, secukinumab was preferred in seven patients, and no malignant progression was observed during the follow-up period. However, their study was not specific to secukinumab, and the start of treatment with secukinumab and follow-up time were not reported [17]. The study with the highest number of patients related to spondyloarthritis included 22 patients and was conducted recently [12]. Similar to our study, the follow-up time in this study by Farina et al. was more prolonged than in previous studies, with a median of 30 months. Tumor recurrence was not observed, and ASDAS scores improved at the last visit compared to the baseline [12]. In our study, in addition to the study by Farina et al., detailed information on tumor stage at diagnosis was provided, and ASDAS CRP and other activity indices, laboratory parameters, and drug retention rates were evaluated. As two different diseases were treated in this patient group, evaluating tumor progression and rheumatologic disease activity with an oncologic–rheumatologic multidisciplinary approach is important.

The results of our study and previous studies on the use of secukinumab in patients with malignant diseases are favorable. Even in two patients with multiple primary tumors and one patient with active disease before treatment with secukinumab, no tumor progression was observed. In our study, one patient with differentiated thyroid carcinoma with active disease received sorafenib in addition to secukinumab for oncologic treatment. Examples of secukinumab treatment in combination with malignancy treatment can be found in case studies in inflammatory arthritis due to immune checkpoint inhibitors [28]. In two patients with active melanoma who were treated with immune checkpoint inhibitors, inflammatory arthritis developed, and they were treated with secukinumab. In that study, by Vincent et al., no tumor progression developed, and the inflammatory arthritis was controlled with secukinumab [28]. In recent years, the emphasis on the pro-inflammatory and pro-mitogenic role of IL17 has paved the way for the combined use of IL17 inhibitors in inflammatory arthritis [17]. IL-17 activates the tumor microenvironment, characterized by proangiogenic and immunosuppressive properties, and induces inflammatory mediators that can lead to cell growth and metastasis [15]. Consistent with these data, the inhibition of IL 17 has been shown to inhibit tumor progression in animal models [16]. Consistent with all these reassuring results, secukinumab is not thought to confer an increased risk of cancer recurrence.

In our study, survival rates were higher with secukinumab treatment compared with phase III clinical trials and real-world studies [29,30,31,32,33,34,35,36]. In the evaluation of previous studies, bionaive patients showed higher drug retention rates compared to patients with bioexperience [34,35,37,38,39,40]. The reason for the higher drug retention rate in our study compared to previous studies may be the higher proportion of bionaive patients due to a history of malignancy in our inclusion criteria. In addition to high drug retention rates, improvement was found in disease activity indices and inflammation markers at the month 12 visit compared to baseline. Furthermore, secukinumab was well tolerated, and no significant side effects were observed in our study. According to our research and previous studies, secukinumab treatment can be used safely to improve disease activity in patients with axSpA and PsA [34,35,37,38,39,40].

Our study offers valuable insights. Nonetheless, some limitations may impact the generalizability of the results, particularly in assessing cancer recurrence, drug survival, and treatment efficacy. Only descriptive statistics could be provided when evaluating neoplasms due to the heterogeneity of the patients’ cancer types. The small number of patients and the group selected due to the study design may limit the survival and treatment efficacy evaluation. In addition, the retrospective design, including only the Turkish population, and the short follow-up period could affect the generalizability of our results. Despite these limitations, the strengths of our study are that it is multicenter, shows real-life data, has a more extended follow-up period compared to many previous studies, and includes the largest cohort.

## 5. Conclusions

Our study found no new tumor development and only one instance of local tumor recurrence in urothelial carcinoma of the urinary bladder treated with secukinumab. Survival rates during treatment were high, and there was an observed improvement in disease activation markers compared to baseline at the month 12 visit. Treatment with secukinumab could be an effective and safe option for axSpA and PsA patients with a history of malignancy. Further real-life studies are warranted in this specific patient group, particularly as neoplasms may become more prevalent with the aging population.

## Figures and Tables

**Table 1:** Demographic and rheumatologic features of the study population at the start of secukinumab treatment.

Male sex *n* (%)	20 (66.7)
BMI kg/m^2^ median (minimum–maximum)	27.1 (21.4–46.9)
Smoking *n* (%)	
Current	4 (13.3)
Previous	8 (26.7)
Never	18 (60)
Alcohol	
Current	4 (13.3)
Previous	1 (3.3)
Never	25 (83.3)
Comorbidity, *n* (%)	
Hypertension	8 (26.7)
Diabetes mellitus	7 (23.3)
Dyslipidemia	5 (16.7)
Cardiovascular disease	2 (6.7)
Chronic obstructive pulmonary disease	1 (3.3)
Hypothyroidism	3 (10)
Multiple sclerosis	1 (3.3)
Chronic renal failure	3 (10)
Spondyloarthritis characteristics	
Diagnosis age of axSpA/PsA years (mean ± std deviation)	42.4 ± 11.5
HLA B27 *n* (%)	17 (56.7)
AxSpA *n* (%)	20 (66.7)
PsA with axial involvement *n* (%)	8 (26.7)
PsA with peripheral involvement *n* (%)	2 (6.6)
Enthesitis *n* (%)	4 (13.3)
Dactylitis *n* (%)	3 (10)
Uveitis *n* (%)	2 (6.7)
Disease duration, years median (minimum–maximum)	12 (1.2–38.1)
Biological therapies	
Bionaive	11(36.7)
Bioexperienced *	19(63.3)
Adalimumab	12 (63.2)
Etanercept	7 (36.8)
Certolizumab	4 (21)
Infliximab	4 (21)
Golilumab	4 (21)
Ustekinumab	1 (5.2)

BMI = body mass index, HLA = human leucocyte antigen, AxSpA = axial spondyloarthritis, PsA = psoriatic arthritis. * Some patients received several different biological therapies before treatment with secukinumab.

**Table 2:** Clinical characteristics of tumors and treatment in our study population.

Patients	Sex	Age at axSpA/PSA Diagnosis	Type of Cancer	Age at Tumor Diagnosis	TNM Staging Classification	Type of Therapy	Time Between Tumor Diagnosis and Treatment with Secukinumab (Months)	Relapse in Cancer During Secukinumab Treatment	Secukinumab Treatment Duration (Months)
1	f	30.6	Papillary thyroid carcinoma	43.5	T1N0M0	Surgery	4.2	No	50.2
2	f	32.1	Papillary thyroid carcinoma	37.8	T1N0M0	Surgery	26.5	No	14.4
3	m	40	Papillary thyroid carcinoma	49	T1N0M0	Surgery	12.2	No	53
4	f	65.9	Papillary thyroid carcinoma	64	T1N1M0	Surgery RAI	79.8	No	58.4
5	f	37	Papillary thyroid carcinoma	44.1	T1N1M0	Surgery	11.3	No	16.5
6	f	34.6	Breast invasive ductal carcinoma	45.9	T1N1M0	Surgery Chemotherapy Radiotherapy Hormonotherapy	59.7	No	7.1
7	m	63	Breast invasive ductal carcinoma	71	T1N0M0	Surgery Radiotherapy Hormonotherapy	118.1	No	32.3
8	f	25.1	Breast invasive ductal carcinoma	49.1	T1N0M0	Surgery Radiotherapy Hormonotherapy	171.7	No	15.3
9	f	45.4	Breast invasive ductal carcinoma	47.4	T1N0M0	Surgery Radiotherapy Hormonotherapy	111.4	No	26.9
10	m	20.9	Renal cell carcinoma	40.1	T1N0M0	Surgery	3.4	No	11.3
11	m	39.3	Renal cell carcinoma	52	T1N0M0	Surgery	2.7	No	17.5
12	m	49.8	Renal cell carcinoma	56.9	T1N0M0	Surgery	14.4	No	37.7
13	m	54.3	Prostate adenocarcinoma	66.3	T2N0M0	Surgery	98.6	No	15.3
14	m	49.4	Prostate adenocarcinoma	62.5	T2N0M0	Surgery	5	No	71.3
15	m	65.8	Prostate adenocarcinoma	68.6	T1N0M0	Surgery	28.6	No	51.4
16	m	47.8	Colon adenocarcinoma	54.4	T4N0M0	Surgery Chemotherapy Radiotherapy	95.4	No	10.6
			Renal cell carcinoma	54.4	T1N0M0	Surgery	95.4	No	
			Papillary thyroid carcinoma	55.1	T1N0M0	Surgery	86.9	No	
17	m	37.2	Colon adenocarcinoma	32.2	T3N1M0	Surgery Chemotherapy	218	No	11.7
18	m	56.6	Lung adenocarcinoma	66.6	T1N0M0	Surgery	18.5	No	46.6
			Basal-cell carcinoma	60.6	T1N0M0	Surgery	90.5	No	
19	m	54	Lung adenocarcinoma	66	T1N0M0	Surgery	59.2	No	18.1
20	m	51.1	Lung squamous-cell carcinoma	65.1	T2N2M0	Surgery Chemotherapy Radiotherapy	28.6	No	12.3
21	m	45.9	Rectum adenocarcinoma	48.9	T1N0M0	Surgery	12.2	No	40.8
22	f	39.9	Uterine endometrioid carcinoma	45.9	T1N0M0	Surgery Brachytherapy	136.7	No	9.2
23	f	39.8	Cervix squamous-cell carcinoma	39.4	T1N0M0	Surgery	105.7	No	23.7
24	f	37.2	Mucinous ovarian carcinoma	46.7	T1N0M0	Surgery	15.4	No	67
25	m	52	Bladder urothelial carcinoma	53.9	T1N0M0	Surgery	17.6	Yes	61.4
26	m	50.3	Jejunoileal gastrointestinal stromal tumors	53.3	T2N0M0	Surgery	25.4	No	15.5
27	m	33	Liposarcoma	41.2	T2N0M0	Surgery Radiotherapy	58.5	No	29
28	f	28.9	Diffuse large B-cell lymphoma	38.3	Stage 2	Chemotherapy	10.4	No	13.2
29	f	32.8	Diffuse large B-cell lymphoma	29.2	Stage 2	Chemotherapy	58.6	No	26.9
30	m	34.8	Differentiated thyroid carcinoma	47.1	T3N1M0	Surgery RAI Sorafenib after recurrence	21.6	No	28.3

T = tumor, N = node, M = metastasis, m = male, f = female, RAI = radioactive iodine.

**Table 3:** Comparison of inflammatory markers and disease activity between secukinumab baseline and 12th-month visit.

	Baseline Visit	12th-Month Visit	*p*-Value
ESR (mm/h)	39.3 ± 20	20 ± 9	<0.001 ^a^
CRP (mg/L)	20.6 (2–73)	5 (2–46)	0.001 ^b^
BASDAI	6.2 (5.4–8.2)	1.8 (1–6.6)	<0.001 ^b^
BASFI	5.5 (3.2–8.1)	1.4 (1–6.9)	<0.001 ^b^
VAS (0–10)	8 (6–10)	2 (0–5)	<0.001 ^b^
ASDAS CRP	4.3 (3.1–5.2)	1.3 (1.1–2.8)	<0.001 ^b^

ESR = erythrocyte sedimentation rate, CRP = C-reactive protein, BASDAI = Bath Ankylosing Spondylitis Disease Activity Index, BASFI = Bath Ankylosing Spondylitis Functional Index, VAS = visual analog scale, ASDAS = Ankylosing Spondylitis Disease Activity Score. ^a^ Paired-samples test; ^b^ Wilcoxon test.

## Data Availability

The data presented in this study are available on request from the corresponding author.
